# Change of ranibizumab-induced human vitreous protein profile in patients with proliferative diabetic retinopathy based on proteomics analysis

**DOI:** 10.1186/s12014-018-9187-z

**Published:** 2018-03-09

**Authors:** Chen Zou, Changjing Han, Minjie Zhao, Jingjing Yu, Lin Bai, Yuan Yao, Shuaixin Gao, Hui Cao, Zhi Zheng

**Affiliations:** 1 Department of Ophthalmology, Shanghai General Hospital, Shanghai Key Laboratory of Ocular Fundus Disease, Shanghai Engineering Center for Visual Science and Photomedicine, No. 100 Haining Road, Shanghai, 200080 China; 2 Department of Ophthalmology, The Second Affiliated Hospital of Xi’an Jiaotong University, Xi’an, 710004 Shaanxi Province China; 3 Department of Ophthalmology, Yixing People’s Hospital, Jiangsu University, No.75 Tongzhenguan Road, Yixing, 214200 Jiangsu China; 4 Department of Ophthalmology, Changshu the 2nd People’s Hospital, Changshu, 215500 Jiangsu China; 5 Public Health, Stanford University, Stanford, CA 94305 USA; 6 National Center for Protein Science Shanghai, Shanghai Institutes for Biological Sciences, Chinese Academy of Sciences, 333 Haike Road, Shanghai, 201210 China

**Keywords:** Proteomics, Proliferative diabetic retinopathy, Ranibizumab

## Abstract

**Background:**

Preoperative treatment of anti-vascular endothelial growth factor (VEGF) agents is extensively used in proliferative diabetic retinopathy (PDR), but the molecular mechanism is not fully understood. The objective of this research is to observe change of protein profile induced by ranibizumab (an anti-VEGF agent) in vitreous humor from PDR patients and reveal the effects of anti-VEGF treatment on PDR.

**Methods:**

A proteomic method was used to identify differentially expressed proteins in vitreous humor. Untreated PDR patients were defined as PDR group, while those who treated with intravitreal injection of ranibizumab (IVR) were defined as IVR. Gene Ontology (GO) annotation and REACTOME pathways were obtained from DAVID Bioinformatics Resources. Intravitreal level of apolipoprotein C-I (APOC1), serpin peptidase inhibitor clade A member 5 (SERPINA5), tissue inhibitor of metalloproteinases (TIMP2), and keratin 1 (KRT1) were determined by enzyme-linked immuno sorbent assay (ELISA).

**Results:**

339 differentially expressed proteins were identified in response to IVR. The most notable GO annotation describes the altered proteins was “innate immune response”. The most notable REACTOME pathway was “platelet degranulation”. ELISA result showed increased level of APOC1, SERPINA5, KRT1 and a decreased level of TIMP2 in PDR group compared with IVR.

**Conclusions:**

In addition to decreasing VEGF level, ranibizumab is associated with change of human vitreous protein profile in patients with PDR, in which the differential proteins are involved in immune response, platelet degranulation, complement activation etc., suggesting that the effects of VEGF are involved in these signaling pathways.

**Electronic supplementary material:**

The online version of this article (10.1186/s12014-018-9187-z) contains supplementary material, which is available to authorized users.

## Background

Diabetic retinopathy (DR) is a progressive disease that leads to vision loss and is the most common chronic complication of diabetes mellitus among working-age adults in developed countries [1]. Vascular endothelial growth factor (VEGF) is a major cytokine playing a central role in mediating microvascular pathology in proliferative diabetic retinopathy (PDR) [2]. As an anti-VEGF agent, ranibizumab is an engineered, humanized, recombinant antibody fragment active against all VEGF-A isoforms and has a shorter half-life than other similar agents [3]. Increasing evidences showed that preoperative anti-VEGF treatment reduces the risk of intraoperative or postoperative bleeding in PDR patients and improves best corrected visual acuity (BCVA) [4–8]. Recently, we found that preoperative intravitreal injection of ranibizumab (IVR) for patients with severe proliferative diabetic retinopathy (PDR) contributed to a decreased risk of postoperative neovascular glaucoma [9], which is among the most serious postoperative complication of PDR. However, anti-VEGF treatment may lead to the risk of fibrosis and tractional retinal detachment (TRD) [10]. Therefore, it is important to explore the mechanism underlying the acts of anti-VEGF treatment agents on PDR such as ranibizumab for not only therapeutic effects but also side effects.

Previous research results showed that intravitreal injection of ranibizumab (IVR) significantly decreased vitreous VEGF level, moreover, downregulated a series of cytokines including interleukin-2 (IL-2), IL-17, intercellular adhesion molecule 1 (ICAM1), and monocyte chemoattractant protein (MCP-1) [11–13]. However, whether ranibizumab causes the change of human vitreous protein profile in PDR patients is still unclear.

To better understand the pathophysiology of PDR and to identify DR-associated risk factors, a proteomics analysis was performed to compare vitreous protein profiles of DM patients with and without development of DR, and some studies results showed that many specific subset of proteins such as inflammation, complement, and coagulation cascade proteins, protease inhibitors, apolipoproteins, immunoglobulins, and cellular adhesion molecules are involved in the pathogenesis of DR [14–17]. In present study, we used a proteomics method to identify differentially expressed proteins in vitreous humor from patients with PDR treated with IVR compared to those from PDR patients without IVR treatment. Our results provided new findings:(1) 339 differentially expressed proteins were identified in vitreous humor from PDR patients in response to IVR; (2) IVR treatment not only decrease intravitreal level of VEGF in PDR patients, but also proteins regulating inflammation, apoptosis, angiogenesis, immune, bleeding and coagulation et al.; (3) APOC1, TIMP2, KRT1 and SERPINA5 may be involved in the development of PDR and in the mechanism of the effects of anti-VEGF treatment.

## Methods

**Fig. 1:**
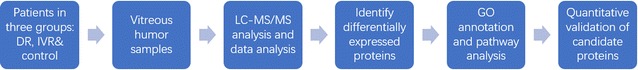
Overall experimental strategy

Overall experimental strategy was described in Fig. 1.

### Patients and sample collection

Diabetic patients were included if they had PDR-related complications such as persistent vitreous hemorrhage more than 1 month in a patient with no history of pan retinal photocoagulation (PRP), vitreous hemorrhage with retinal detachment according to B-Scan ultrasonography, and macula-involving or macula-threatening TRD. The research was approved by the hospital’s research ethics committee. Informed consent was obtained from each patient, and the experimental procedures followed the tenets of the Declaration of Helsinki. Patients’ rights to privacy were protected in our study. All the patients underwent 25G pars plana vitrectomy (PPV). PDR patients were randomly divided to two groups. Those who had been preoperatively treated with IVR were defined as IVR group (n = 9). Intravitreal injection of ranibizumab (0.5 mg, Novartis) was performed 5–7 days before PPV. While those who were not treated with IVR were defined as the PDR group (n = 8). Non-diabetic patients with idiopathic macular hole (iMH) made up the control group (n = 9). Exclusions were history of photocoagulation treatment; history of ocular trauma or surgery; vasculopathy besides PDR, including retinal vein obstruction or retinal vasculitis; other ocular disease including neovascular glaucoma, age-related macular degeneration, or rhegmatogenous retinal detachment; systemic disease other than diabetes mellitus, including autoimmune disease or high blood pressure. The level of fasting blood glucose (FBG) of all patients was determined preoperatively, while glycosylated hemoglobin (HbA1C) was determined for PDR and IVR group patients but not MH patients to avoid over-examination. During PPV, 0.2–0.3 mL of undiluted vitreous humor was removed from the posterior segment using the 25G vitreous cutter. The vitreous humor was centrifuged for 10 min at 4 °C and 15000 rpm; then the supernatant was stored in liquid nitrogen. Baseline information was obtained from the medical records included including age, gender, lens status, laboratorial data, presence of preoperative vitreous hemorrhage and TRD.

### Sample preparation

Protein contents was measured by using BCA kit (beyotime, Beijing, China) according to the manufacturer’s instructions. To 1 volume of cold sample solution, 1/3 volume of 100% (w/v) TCA (6.1 N, Sigma,) was added and mixed well to give a final TCA concentration of 20–25%. The solution was placed on ice for 4 h in a cold room (4 °C) then centrifuged for 30 min at 4 °C. The supernatant was aspirated with a gel loading tip, leaving 5–10 μL in the tube so as not to disturb the pellet. The pellet was washed twice with 500 μL ice-cold acetone. After each wash, it was centrifuged for 10 min and dried using speed vacuum or air for 1–2 min. The washed protein pellet was dissolved in 8 M urea with 100 mM Tris–HCl at pH 8.5. To reduce disulfide bonding, TCEP was added (final concentration, 5 mM) to the solution and incubated for 20 min at room temperature. The residue was alkylated using iodoacetamide (final concentration, 10 mM) for 15 min at room temperature. The protein mixture was diluted four times and digested with trypsin (Promega, Beijing, China) at a concentration of 1:100 w/w.

### LC–MS/MS analysis and data analysis

This procedure was performed in National Center for Protein Science Shanghai according to published researches [18, 19] (See detailed description in Additional file 1). Normalized spectral abundance factor (NSAF) which was firstly proposed by Florens et al. [20] was used to evaluate the relative protein contents base on the spectrum counts. This method uses protein length to normalize spectral count (SC) for improving the accuracy. For each protein in a given database, the NSAF score is: ${\text{NSAF}}_{\text{N}} = \frac{{{\text{SN}}/{\text{LN}}}}{{\mathop \sum \nolimits_{i = 1}^{n} si/Li}}$, where: N is protein index; SN is the number of peptide spectra matched to the protein; LN is the length of protein N; n is the total number of proteins in the input database. Refer to the method reported by Wisniewski et al. [19], quantifiable proteins in the analysis of samples were defined as those identified at least 50% in at least one type of sample (PDR, IVR and control). Therefore, in case that proteins with low abundance close to the detection limit but identified in less than 50% of the sample were defined as undetected. DAVID Bioinformatics Resources 6.8 (https://david.ncifcrf.gov/tools.jsp) was used to obtain Gene Ontology (GO) annotation and REACTOME pathways.

### Validation of proteomic analysis

To confirm changes in the intravitreal level of specific candidate proteins, ELISA was performed on the vitreous humor samples using ELISA kits (CUSABIO) according to the manufacturer’s instructions. Protein contents was calculated based on the OD value and dilution ratio.

### Statistical methods

SPSS (version 17) and SAS (version 9) were used for statistical analysis. All continuous variables exhibited a typical normal distribution, as tested by the Shapiro–Wilk method. Comparisons among groups were conducted using one-way ANOVA. Multiple test correction was performed by SNK method. The level of statistical significance was *P* < 0.05.

## Results

Total twenty-six patients were recruited in our study, including 8 untreated PDR patients, 9 PDR patients treated with IVR and 9 non-diabetic patients. After proteome profiling, differentially expressed proteins were identified between PDR and control group, PDR and IVR group, respectively. Then, DAVID Bioinformatics Resources was applied to do GO annotation and pathway analysis. After these, ELISA was used to determine and confirm the intravitreal levels of candidate proteins.

### Baseline findings

Demographic and experimental data for all patients are summarized in Table 1. There were no significant differences in demographic and experimental data among groups except for a statistically significant difference in fasting plasma glucose between the control group and the other two groups (*P* < 0.01).

**Table 1:** Demographic and laboratorial data at baseline

Characteristic	Control (n = 9)	PDR (n = 8)	IVR (n = 9)	*P* value
Age (years)	53.9 ± 12.5	47.5 ± 10.7	49.1 ± 8.6	> 0.05
Gender (male/female)	4/5	4/4	4/5	> 0.05
FBG(mmol/l)	6.0 ± 0.7	9.7 ± 0.9*	9.6 ± 3.3*	< 0.01
HbA1C(%)	–	9.1 ± 1.3	8.8 ± 1.3	> 0.05
Lens status (phakic/pseudophakic)	4/5	3/5	4/5	> 0.05
Indication for surgery				
VH	–	8 (100%)	9 (100%)	> 0.05
TRD	–	3 (37.5%)	3 (33.3%)	> 0.05

*VH* vitreous hemorrhage, *TRD* tractional retinal detachment

*Statistically significant difference with control group

There were no significant differences in demographic and experimental data among groups except for a statistically significant difference in fasting plasma glucose between the control group and the other two groups (*P *< 0.01)

### Proteomic analysis of proteins that are differentially expressed in PDR

**Fig. 2:**
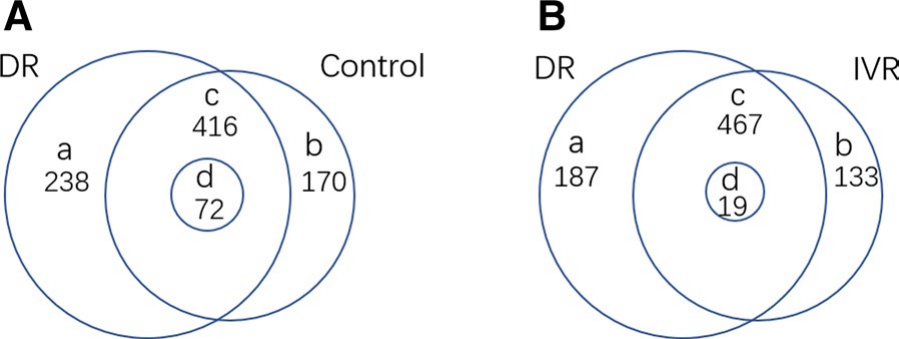
**A** Proteins detected in (a) only PDR group, (b) only control group, (c) both groups, (d) differentially expressed among the proteins detected in both groups. **B** Proteins detected in (a) only PDR group, (b) only IVR group, (c) both groups, (d) differentially expressed among the proteins detected in both groups

As shown in Fig. 2A, proteomic analysis identified 654 and 586 intravitreal proteins by LC/MS–MS in PDR and control group (See Additional file 2: Table S1 for a list of detected proteins in each group). Among these proteins, 238 were detected only in PDR group and 170 were detected only in control group. 416 proteins were detected in both groups. Among the 416 proteins, 72 proteins were differentially expressed. (*P* < 0.05) (See Additional file 2: Table S2 for a list of 72 differentially expressed proteins).

### Proteomic analysis of intravitreal proteins that are differentially expressed in response to IVR

As shown in Fig. 2B, 654 proteins were detected in PDR group and 600 proteins were detected in IVR group (See Additional file 2: Table S1 for a list of detected proteins in each group). Among these proteins, 187 were detected only in PDR group (Additional file 2: Table S3) and 133 were detected only in IVR group (Additional file 2: Table S4). 467 proteins were detected in both groups. Among the 467 proteins, three proteins were significantly up-regulated and 16 proteins (including VEGFA) were significantly down-regulated in IVR group compared to PDR. (*P*<0.05) (Additional file 2: Table S5). Therefore, a total of 339 proteins were differentially expressed. Among these differentially expressed proteins, 203 proteins were decreased (including 187 proteins detected only in PDR group and 16 decreased proteins in IVR group) and 136 proteins were increased (including 133 proteins detected only in IVR group and three increased proteins in IVR group) in response to IVR.

### GO annotation and pathway analysis of proteins altered in response to IVR

To obtain a functional overview of identified proteins by LC–MS/MS, GO annotations and REACTOME pathways describe the differentially expressed proteins in response to IVR were obtained from DAVID Bioinformatics Resources. Among the 339 differentially expressed proteins, 69 decreased proteins and 42 increased proteins in response to IVR were found in database and submitted as gene list.

#### GO annotation and pathway analysis of the total differentially expressed proteins

**Fig. 3:**
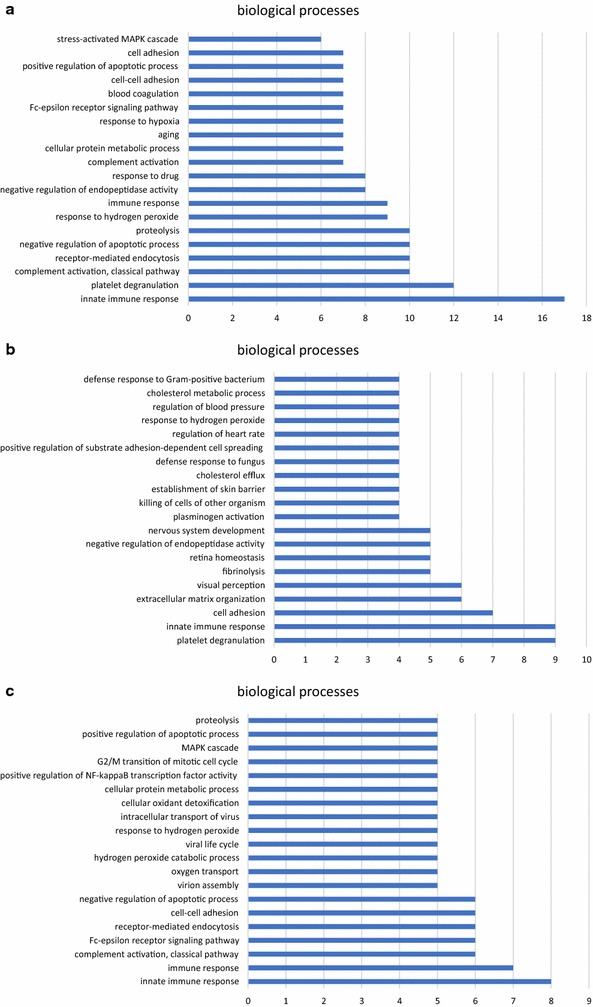
GO annotation of the main altered proteins in response to IVR. **a** the most notable 20 of 174 GO annotations of all the differentially expressed proteins, **b** the most notable 20 of 87 GO annotations of the decreased proteins, **c** the most notable 20 of 102 GO annotations of the increased proteins

**Fig. 4:**
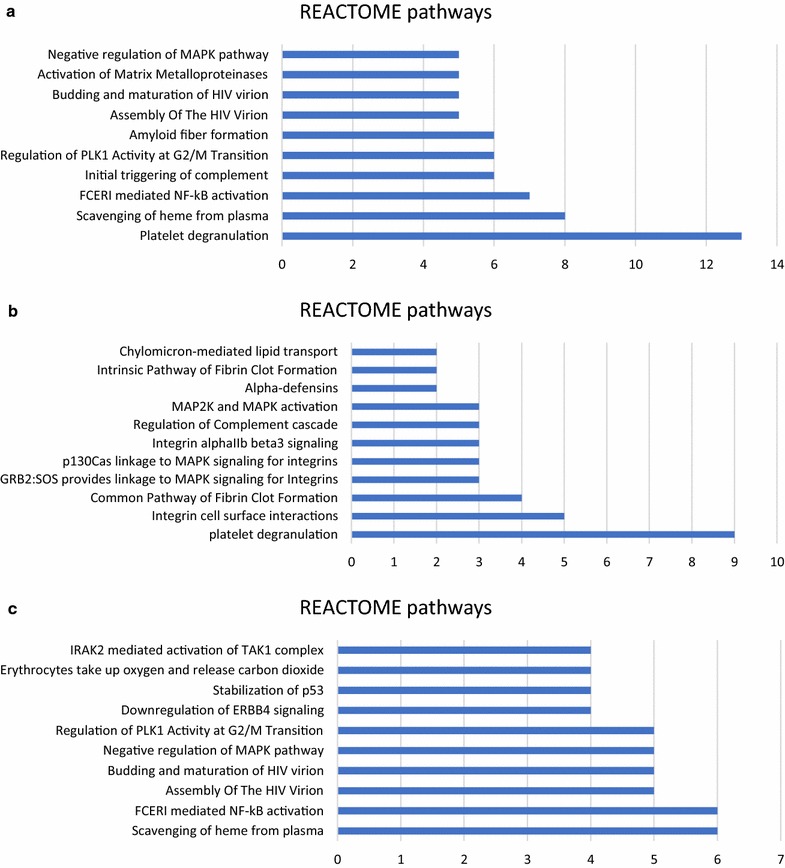
REACTOME pathway analysis of the altered proteins in response to IVC. **a** The most notable 10 of 148 REACTOME pathways of all the differentially expressed proteins. **b** REACTOME pathways of the decreased proteins. **c** the most notable 10 of 139 REACTOME pathways of the increased proteins

254 GO annotations and 148 REACTOME pathways were found to generalize the function and localization of all the differentially expressed proteins. The proteins involved in biological processes were further classified into 174 different subcategories. Figure 3a showed the most notable 20 annotations in biological processes. The highest number of proteins were involved in “innate immune response” (17 proteins), followed by “platelet degranulation” (12 proteins), “complement activation” (10 proteins), “receptor-mediated endocytosis” (10 proteins), “negative regulation of apoptotic process” (10 proteins), and “proteolysis” (10 proteins). Proteins involved in molecular functions were classified into 38 different categories, with the most notable of “protein binding” (68 proteins). Proteins involved in cellular components were classified into 42 categories, with the most notable of “extracellular exosome” (76 proteins). Figure 4a showed the main 10 pathways. The most notable pathway was “Platelet degranulation” (13 proteins), followed by “Scavenging of heme from plasma” (8 proteins) and “FCERI mediated NF-kB activation” (7 proteins).

#### GO annotation and pathway analysis of decreased proteins

143 GO annotations and 11 REACTOME pathways were found to generalize the function and localization of the decreased proteins in response to IVR. The proteins involved in biological processes were further classified into 87 different categories. Figure 3b showed the most notable 20 annotations in biological processes. The highest number of proteins were involved in “platelet degranulation” (AHSG, FGA, FGB, FGG, ITIH3, RARRES2, SOD1, TF and VEGFA) and “innate immune system” (CHGA, C2, CFI, DEFA1, DEFA3, FGA, FGB, IGLC7 and KRT16). The remaining proteins were linked to different activities such as cell adhesion, extracellular matrix organization, visual perception, fibrinolysis, retina homeostasis et al. Proteins involved in molecular functions were classified into 24 different categories, with the most notable of “protein binding” (37 proteins). Proteins involved in cellular components were classified into 32 categories, with the most notable of “extracellular exosome” (43 proteins). Figure 4b showed all the eleven pathways, the most notable of which was “platelet degranulation” (AHSG, FGA, FGB, FGG, ITIH3, RARRES2, SOD1, TF and VEGFA), followed by “integrin cell surface interactions” (COL18A1, FGA, FGB, FGG and SPP1) and “common pathway of fibrin clot formation” (FGA, FGB, FGG and SERPINA5).

#### GO annotation and pathway analysis of increased proteins

137 GO annotations and 139 REACTOME pathways were found to generalize the function and localization of the increased proteins in response to IVR. The proteins involved in biological processes were further classified into 102 different categories. Figure 3c showed the most notable 20 annotations in biological processes. The highest number of proteins were involved in “innate immune response” (C1QA, C1R, C4BPA, IGHA2, RPS27A, UBA52, UBB and UBC) and “immune response” (C1R, FTH1, IGHA2, IGHV3-53, IGLV1-47, PNP, and SEMA7A). The remaining proteins were linked to different activities such as complement activation, Fc-epsilon receptor signaling, receptor-mediated endocytosis, cell adhesion, negative regulation of apoptotic process et al. Proteins involved in molecular functions were classified into 18 different categories, with the most notable of “protein binding” (31 proteins). Proteins involved in cellular components were classified into 17 categories, with the most notable of “extracellular exosome” (33 proteins). Figure 4c showed the main 10 pathways, the most notable of which was “Scavenging of heme from plasma” (HBA1, HBA2, HBB, IGHA2, IGHV3-53 and IGLV1-47).

### Validation of 4 candidate proteins by ELISA

**Fig. 5:**
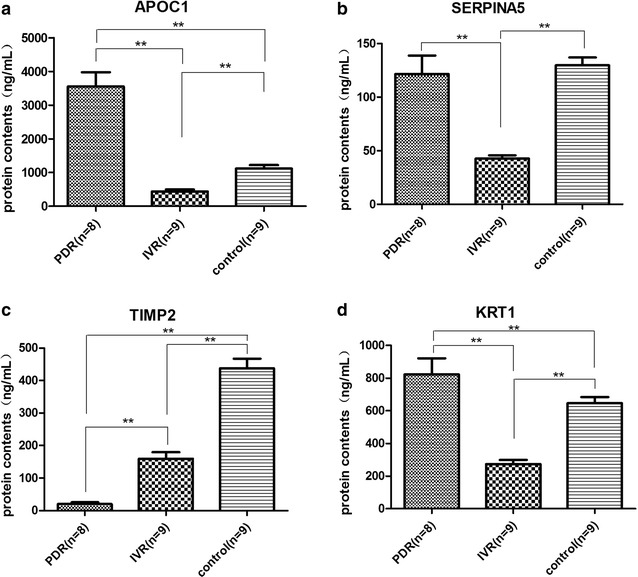
Intravitreal level of **a** APOC1, **b** SERPINA5, **c** TIMP1, **d** KRT1 was confirmed by ELISA. **P < 0.01 between each group

We searched the main function of differentially expressed proteins on Pubmed (https://www.ncbi.nlm.nih.gov/pubmed/). According to the function related to inflammation, angiogenesis, apoptosis, fibrosis and tumor growth, which are closely associated with either the development of DR or VEGF signaling, we were interested in following candidate proteins: APOC1, SERPINA5, TIMP2, and KRT1. These proteins are seldom researched in the pathology of DR. We performed ELISA to confirm the relative abundances of these four candidate proteins. The differences in expression were generally consistent with the results of LC–MS/MS. APOC1 and KRT1 expression were greatly increased in the PDR group compared to the IVR and control groups (Fig. 5a, d). SERPINA5 expression in PDR group was not significantly different from the control group, but was much greater than in the IVR group (Fig. 5b), while the expression of TIMP2 was weak or undetectable in the PDR group (Fig. 5c).

## Discussion

Previous proteomic studies of human vitreous humor have identified a number of proteins that are differentially expressed in rhegmatogenous retinal detachment, proliferative vitreoretinopathy, intravitreal inflammation, diabetic macular edema, idiopathic epiretinal membranes, or particularly diabetic retinopathy [15, 21–25]. In this study, we used a LC–MS/MS based proteomics study to visualize the change of ranibizumab-induced human vitreous protein profile in patients with PDR. Normalized spectral abundance factor (NSAF) was used to evaluate the relative protein abundance base on the spectrum counts, which has been demonstrated to be an effective quantitative proteomics approach [26–31].

We first identified 480 differentially expressed proteins between the PDR and control groups, as similar as that in the previous results from Kim et al. [14, 15]. Further, for the first time, our results have demonstrated that ranibizumab can induce the change of total protein profile (more than 300 differentially expressed proteins) in human vitreous of proliferative diabetic retinopathy patients after IVR treatment. We first submit all the 339 differentially expressed as gene list into DAVID Bioinformatics Resources. It is found that these proteins mainly play roles in innate immune response, platelet degranulation, complement activation, endocytosis, apoptosis, proteolysis and heme scavenging et al., suggesting that the effects of VEGF are involved in these signaling pathways. Therefore, the therapeutic effects of ranibizumab on PDR are not only decreasing VEGF but also regulating the signaling pathways. Next, we submit the increased proteins and decreased proteins of the 339 differential proteins separately into the database. Our results showed that “platelet degranulation” mainly described the decreased proteins in response to IVR but not the increased ones. Platelets contains a number of distinguishable storage granules including alpha granules, dense granules and lysosomes. The homeostasis between platelet aggregation and degranulation which protect against vascular damage was found to be disturbed in diabetes [32]. Platelet degranulate can release a series of polypeptide or small molecules such as fibrinogen, growth factors, protease inhibitors that supplement thrombin generation at the site of injury. It was suggested that treatment with IVR can regulate blood coagulation and vascular damage by inhibiting the degranulation of platelet and may have protective effects on PDR.

Besides the most notable pathway “platelet degranulation”, many decreased proteins also involved in “integrin cell surface interactions” pathway. Integrins are the receptors that mediate cell adhesion to ECM, which is a network of macro-molecules that underlies all epithelia and endothelia and that surrounds all connective tissue cells. For example, α2β1 integrin is a platelet receptor and is associated with DR [33]. High glucose can also increase apoptosis of pericytes via α3β1 and αvβ5 signaling [34, 35]. Integrin α4 is a mediator of leukocyte adhesion to the vascular endothelium of the diabetic retina, which results in endothelial injury, blood-retina barrier breakdown, and capillary nonperfusion [36]. Therefore, integrins are very important system inducing inflammation, apoptosis, angiogenesis and so on in the pathology of DR. Our data showed that many decreased proteins in response to IVR are involved in integrin cell surface interactions, suggesting that IVR treatment may have protective effects on PDR by the inhibition of integrin interaction.

Immune response plays critical roles in DR through the presence of antipericyte and antiendothelial cell autoantibodies and the abnormal expression of T cells [37–39]. Our data showed that 9 decreased proteins and 8 increased proteins were all involved in “innate immune response”. Moreover, there were 7 increased proteins involved in “immune response” annotation, but there was no decreased protein involved in this annotation. Thus, although immune response is important when generalizing the differentially expressed proteins in response to IVR, it was hard to judge whether IVR bring positive or negative impact on immune system on the development of PDR. Further quantitative analysis need to be performed on the proteins mainly involved in immune response in PDR.

Among the differentially expressed proteins, we were particularly interested in four proteins: APOC1, KRT1, SERPINA5, and TIMP2. These proteins play critical roles in lipoprotein metabolism, inflammation, angiogenesis, cellular growth, and cytoskeletal integrity, which are all closely associated with development of DR, however, the role of these proteins in the pathogenesis of DR is unclear. In our study, we performed ELISA and the results were generally consistent with those obtained from LC–MS/MS. These results suggest that the proteins may be important in the pathogenesis of DR.

The APOC1 gene plays a central role in lipoprotein and cholesterol metabolism [40, 41]. Moreover, in mice that overexpress APOC1, levels of IL-6 and IL-1 beta are elevated, and in lung cancer patients. APOC1 levels are positively correlated with IL-6 in the serum [42], suggesting that APOC1 is a pro-inflammation cytokine. TIMP2 is in the family of tissue inhibitors of metallopeptidases, which is known to be an anti-angiogenesis protein through inhibition of matrix metallo proteinases or via α3β1 integrin-mediated binding of the N-terminal domain of TIMP2 to endothelial cells [43]. The SERPINA5 gene is a member of the plasma serine protease inhibitor family [44], which is a potent inhibitor of activated protein C (APC), an anti-inflammation protein [45], suggesting that it has a pro-inflammatory effect. Keratins are the basic materials of intermediate filaments, and thus are essential to maintaining cytoskeletal integrity [46]. Recent studies have shown that KRT1 also has effects on the activity of kinases, such as protein kinase C and SRC, which play important roles in inflammation and apoptosis [47]. Moreover, it is detected in endothelial cells, where it binds to kininogen [48]. KRT1 expression in human umbilical vein endothelial cells increases in response to oxidative stress and activates the lectin complement pathway via mannose-binding lectin binding to KRT1 [49]. Elisa results quantitatively validate the change of intravitreal level of APOC1, TIMP2, SERPINA5 and KRT1, suggesting that these proteins may be associated with the pathology of DR and the mechanisms of the effect of IVR.

## Conclusions

In summary, we used proteomic methods to identify differentially expressed proteins in the vitreous humor from diabetic retinopathy patients. 480 proteins were differentially expressed in the vitreous humor from PDR patients compared with non-diabetic iMH patients. 339 proteins were differentially expressed in the vitreous humor from PDR patients treated with IVR compared to those who didn’t treated with IVR. Moreover, anti-VEGF treatment can not only decrease the level of VEGF but also regulate inflammation, apoptosis, angiogenesis, immune, bleeding and coagulation et al. in vitreous humor from PDR patients. Importantly, SERPINA5, APOC1, TIMP2, and KTR1 were confirmed to exhibit differential expression, and may may be associated with the pathogenesis of DR and the effects of anti-VEGF treatment. The identification of the key molecules and pathways are critical for the development of new therapeutic molecules and for the treatment of PDR and may help us to understand the mechanism of anti-VEGF treatment of PDR.

## Additional files

**Additional file 1.** A supplementary description of methods for LC–MS/MS analysis and data analysis.

**Additional file 2.** Protein list detected by LC-MS/MS.

## Abbreviations

VEGF: vascular endothelial growth factor
PDR: proliferative diabetic retinopathy
IVR: intravitreal injection of ranibizumab
APOC1: apolipoprotein C-I
SERPINA5: serpin peptidase inhibitor clade A member 5
TIMP2: tissue inhibitor of metalloproteinases
ELISA: enzyme-linked immuno sorbent assay
DR: diabetic retinopathy
BCVA: best corrected visual acuity
TRD: tractional retinal detachment
ICAM-1: intercellular adhesion molecule 1
MCP-1: chemoattractant protein 1
PRP: pan retinal photocoagulation
GO: gene ontology
PPV: pars plana vitrectomy
FBG: fasting blood glucose
NSAF: normalized spectral abundance factor
iMH: idiopathic macular hole
