# Supplementary material for: Change of ranibizumab-induced human vitreous protein profile in patients with proliferative diabetic retinopathy based on proteomics analysis
Source: Clin Proteomics. 2018 Mar 9;15:12. doi: 10.1186/s12014-018-9187-z (PMC5844103; doi:10.1186/s12014-018-9187-z)
Supplement: Supplementary file 1 — Additional file 1. A supplementary description of methods for LC–MS/MS analysis and data analysis. [file 12014_2018_9187_MOESM1_ESM.docx]

**LC-MS/MS analysis**

A C18 column (100 μm ID, 15 cm length) packed with Aqua C18 reverse phase material (Phenomenex) was placed inline with a PROXEON EASY-nLC1000 liquid chromatography system (ThermoFisher, San Jose, CA). Mobile phases A and B were 0.1% formic acid in water and 0.1% formic acid in acetonitrile, respectively. After loading, the digested peptides were separated using the following gradient: 0–5 min, 0–3% B; 5–150 min, 3–30% B; 150–166 min, 30–100% B; 166–180 min, 100% B. The flow rate was set to 300 nL/min. Peptides eluted from the LC column were directly electrosprayed into an Orbitrap Elite mass spectrometer (ThermoFisher, San Jose, CA) with the application of a distal 1.8 kV spray voltage. A cycle of 1 full-scan MS spectrum (m/z 300–1800) was acquired followed by 20 data-dependent MS scans, sequentially generated for the 20 most intense ions in the full MS spectrum at a 30% normalized collision energy. The number of microscans for both MS and MS/MS scans was one and the maximum ion injection times were 200 and 50 ms, respectively. The dynamic exclusion duration was 60 s. MS scan functions and HPLC solvent gradients were controlled by the Xcalibur data system (ThermoFisher).

**Data Analysis**

Protein analysis was performed using the Integrated Proteomics Pipeline, IP2 (Integrated Proteomics Applications, Inc., San Diego, CA). MS/MS spectra were extracted from raw files using RawXtract 1.9.9.1 (http://fields.scripps.edu/downloads.php), and were searched with the ProLuCID algorithm against the Uniprot human database (http://www.uniprot.org/) within IP2 (MS1: 7ppm, MS2: 0.05 Da). To accurately estimate peptide probabilities and false discovery rates, we used a decoy database containing the reverse sequences of all proteins appended to the target database. The search space included all fully tryptic and half-tryptic peptide candidates that fell within the mass tolerance window. Carbamidomethylation (+57.02146 Da) of cysteine was considered a static modification. The database search results were assembled and filtered using the DTASelect program. Normalized spectral abundance factor (NSAF) was used to evaluate the relative protein contents base on the spectrum counts. The protein detected in less than 50% of the sample was defined to undetected protein.
